# Vitamin D_3_ Is Not a Limiting Nutrient Regarding Growth Performance and Tibia Parameters in the Rearing Period of Laying Hens Bred for High Laying Performance Compared to Non‐Selected Resource Populations

**DOI:** 10.1111/jpn.14104

**Published:** 2025-01-27

**Authors:** Mareike Kölln, Jana Frahm, Ingrid Halle, Liane Hüther, Jeannette Kluess, Henrieke Meyer‐Sievers, Lars Schrader, Steffen Weigend, Sven Dänicke

**Affiliations:** ^1^ Friedrich‐Loeffler‐Institut, Federal Research Institute for Animal Health, Institute of Animal Nutrition Braunschweig Germany; ^2^ Friedrich‐Loeffler‐Institut, Federal Research Institute for Animal Health, Institute of Animal Welfare and Animal Husbandry Celle Germany; ^3^ Friedrich‐Loeffler‐Institut, Federal Research Institute for Animal Health, Institute of Farm Animal Genetics Neustadt Germany

**Keywords:** bone, performance, purebred laying hens, rearing, vitamin D_3_

## Abstract

Bone damages in laying hens are of great concern in poultry farming. Besides various risk factors like housing systems or nutrient supply during egg production, it has often been hypothesized that genetically high‐performing laying hens may be more prone to bone damages. The relevance of dietary support during the rearing period of pullets for optimal bone development has been little addressed so far. In the present study, an increasing dietary vitamin D_3_ content within EU legislation was tested during the first 12 weeks of life in two high and two moderate‐performing pullet lines (white and brown layer lines). For this purpose, a total of 940 chickens of both sexes were housed at the Institute of Animal Welfare and Animal Husbandry (Friedrich‐Loeffler‐Institut, Germany). The three experimental diets differed only regarding the added vitamin D_3_ amount (300/1000/3000 IU Cholecalciferol/kg diet). After every 4 weeks, randomly chosen animals per genotype and dietary treatment were slaughtered for dissection. Serum 25(OH)‐Vitamin D_3_ concentrations reflected the dietary treatment. Body weight differed regarding genotype. No effect of dietary vitamin D_3_ content as a single influence factor on bone parameters like breaking strength, bone dimensions or mineral content could be shown, but age, sex and genotype had impacts and influenced traits in an interactive manner. Therefore, during the first 12 weeks of the rearing period of layer pullets, the different dietary vitamin D_3_ contents did not influence performance or bone parameters in the four genetically diverse purebred layer lines. Adjusted dietary vitamin D_3_ recommendations for pullets depending on genetically predetermined egg‐laying performance do not appear to be necessary if dietary vitamin D_3_ contents are within EU legislation.

## 1 Introduction

Feeding of laying hens places high demands on energy and nutrient supply. In particular, the requirements for calcium/vitamin D during egg (shell) production are of special interest due to skeletal metabolic challenges (Whitehead and Fleming 2000). Previous research suggests that skeletal health, particularly keel bone fractures, is one of the major welfare problems in laying hens (FAWC 2010, 2013). Skeletal health is – among other factors – influenced by the genetic origin of the animals, although the importance of recommended nutrient levels (e.g., calcium, phosphorus, vitamin D) cannot be neglected (Fleming et al. 2006; Gebhardt‐Henrich and Fröhlich 2015; Raymond et al. 2018). Considering that not only high but also moderate performing genotypes are used in egg production (e.g., organic farming), and that white egg layers differ from brown egg layers in terms of bone stability parameters (Habig and Distl 2013), a critical view of the recommended nutrient levels and/or the examination of the adaptive capacity of these different genotypes is necessary.

Vitamin D, an important nutrient for calcium and bone metabolism, is a valuable parameter for this consideration: Calcium is also one of the main elements of the structural bone (Leeson and Summers 2001). During the laying period, calcium reserves often decrease in laying hens due to different reasons (e.g., availability of calcium, Leeson and Summers 2001; Whitehead and Fleming 2000). Although many efforts have been made in adjusting housing, breeding and feeding parameters of laying hens, this challenge still remains even under modern farming conditions (Whitehead and Fleming 2000; Rentsch et al. 2024). With the onset of the laying period in modern laying hens, the bone development of the young animals is not yet complete, which poses a challenge in meeting the requirements of both metabolic processes, bone formation and eggshell production (Buckner et al. 1948; Leeson and Summers 2001; Jansen et al. 2020; Habig et al. 2021; Thøfner, Dahl, and Christensen 2021). It has been suggested that genetic changes to high‐performing lines with more eggs laid per hen may have been achieved at the expense of other health parameters, such as bone stability (Habig et al. 2017). Thus, high‐producing layer lines may be more susceptible to bone fractures than low‐producing lines (Budgell and Silversides 2004), although other studies have found no evidence of this (Dunn et al. 2021). The fact that the timing of the first egg laying has an effect on (keel) bone health (Gebhardt‐Henrich and Fröhlich 2015) also underlines the importance of genetic origin for bone health, as this maturation process differs between different genotypes, with an earlier onset in high producing layer lines (Thiruvenkadan, Panneerselvam, and Prabakaran 2010; Jansen et al. 2020).

Among other bone damages, osteoporosis, a multicausal phenomenon, has been recognized as a serious problem in laying hens for decades (Thorp 1994; Nasr, Nicol, and Murrell 2012; Bryden et al. 2021). Meeting the nutritional requirements of laying hens with commercial complete feeds, therefore, seems to be insufficient to prevent bone damages (Whitehead and Fleming 2000). Due to the prominent role of vitamin D in calcium metabolism (National Research Council [NRC] 1994), an investigation of different dietary vitamin D_3_ levels in young pullets before sexual maturation (purebred layer lines, white and brown egg layers) of both sexes and different genetic performances seems appropriate to further understanding of this issue. The following evaluation of the laying hens is not part of the study presented here.

The hypothesis for the present study was that the minimum recommended dietary vitamin D_3_ content of the GfE Gesellschaft für Ernährungsphysiologie Ausschuss für Bedarfsnormen (1999; 250 IU Vit. D/kg dry matter [DM] for chicks and pullets up to 17 weeks of life) is not sufficient for high performing modern laying hens and their pullets. The current maximum legal content is 3200 IU vitamin D_3_/kg diet (88% DM content; Commission Implementing Regulation EU 2019/849), which should be considered practically relevant. However, different performance levels of the animals may result in different requirements regarding vitamin D_3_ supply.

The present study investigated the effects of graded dietary vitamin D_3_ supplementation on growth performance and various tibia bone parameters of male and female purebred chickens (‘laying lines’), differing in genotype and performance (WLA/R11: high/low performing white layers; BLA/L68: high/low performing brown layers) during 12 weeks of rearing. This study was part of a study investigating the adaptability of high and low performing laying hens to different environmental conditions throughout their entire life. In this substudy, the focus was on the rearing period.

## 2 Materials and Methods

### 2.1 Ethical Note

The study complies with the German Animal Welfare Act, and all procedures have been reviewed and approved by the relevant authorities (Lower Saxony State Office for Consumer Protection and Food Safety, LAVES, Germany; file number 33.92 42502‐04‐13/1186 and 33.19 42502‐04‐15/1816).

### 2.2 Experimental Design and Housing

**Figure 1:**
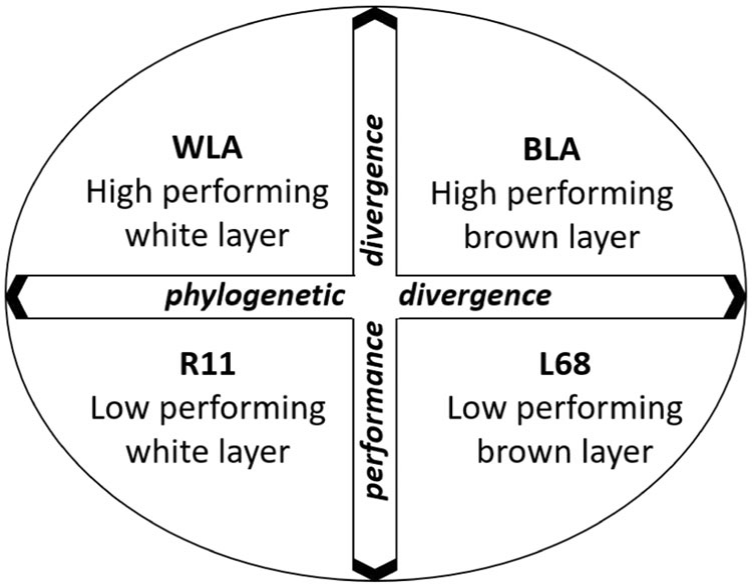
Experimental design according to Lieboldt et al. (2015), modified.

The used experimental poultry model was previously described by Lieboldt et al. (2015). In this model, four purebred layer lines, differing in genotype and performance, were studied (Figure 1).

WLA and BLA are chickens from two high‐performance commercial laying lines (approx. 320 eggs/year; Jansen et al. 2020), which originally came from the breeding program of Lohmann Breeders GmbH in 2012. Since then, they have been kept in a conservation breeding scheme at the Institute of Farm Animal Genetics, Friedrich‐Loeffler‐Institut. In comparison, R11 and L68 are two lower‐performing genotypes kept as resource populations at the same institute with an egg‐laying performance of about 200 eggs/year (Jansen et al. 2020). While WLA and R11 are two white layer lines of White Leghorn, BLA (Rhode Island Red) and L68 (New Hampshire) are brown layer lines.

A total of 940 animals were available for this study. At hatching, 40 animals (10 per genotype) were sacrificed to obtain comparative data for subsequent measurements. The remaining chicks were allocated to 12 groups (4 × 3 factorial design, with 4 genotypes and 3 diets, Table 1) for 12 weeks of rearing. Each group consisted of 5 replicates (5 pens/group). In each 1.05 m^2^ pen, 15 chickens (10 females and 5 males) were housed on a mixture of wood shavings and straw.

**Table 1:** Overview of experimental design.

Genotype	Group	Vit. D_3_‐content of complete diet (IU/kg as fed)	Pens (replicates)/group	Number of animals
WLA	1	300	5	*n* = 75
	2	1000	5	*n* = 75
	3	3000	5	*n* = 75
BLA	4	300	5	*n* = 75
	5	1000	5	*n* = 75
	6	3000	5	*n* = 75
L68	7	300	5	*n* = 75
	8	1000	5	*n* = 75
	9	3000	5	*n* = 75
R11	10	300	5	*n* = 75
	11	1000	5	*n* = 75
	12	3000	5	*n* = 75

Feed and water (nipple drinkers, water from the public supply) were offered ad libitum.

The diets (2‐phase feeding, based on soybean meal and cereals, Table 2) differed only regarding the added vitamin D_3_ content (300/1000/3000 IU/kg, SUNVIT D_3_ 500,000 IU/G, synonym cholecalciferol, Sunvit GmbH, Bardowick, Germany). The starter diets were fed from Weeks 1 to 8 and the pullet diets from Weeks 9 to 12. The diets were formulated to meet the nutrient requirements (NRC 1994; GfE Gesellschaft für Ernährungsphysiologie Ausschuss für Bedarfsnormen 1999). Vitamin D_3_ was added as a feed additive, while the premix for other vitamins and trace elements did not contain vitamin D_3_.

**Table 2:** Diet composition (in % as fed).

Experimental diet	Chickens (Weeks 1–8)			Pullets (Weeks 9–12)		
Vit. D_3_ content (IU/kg)	300	1000	3000	300	1000	3000
Maize	45.75			22.40		
Soybean meal	21.57			13.27		
Wheat	20.00			30.00		
Wheat bran	6.22			3.40		
Grass meal	0			1.66		
Triticale	1.71			25.00		
Soy oil	0.20			0.20		
Dicalcium phosphate	1.96			1.54		
Calcium carbonate	1.03			0.96		
Sodium chloride	0.34			0.41		
Lysine‐HCl	0.08			0		
DL‐Methionine	0.14			0.09		
L‐Threonine	0			0.05		
L‐Tryptophan	0			0.02		
Premix	1.00^a^			1.00^b^		
Vitamin D_3_ (IU/kg)	300	1000	3000	300	1000	3000

^a^ Premix (for chicken diets) provides per kg diet: 0.84% crude ash; 0.21% Ca; 0.12% Na; 32 mg 3b103 (Iron (II) sulphate monohydrate); 12 mg 3b405 (Copper (II) sulphate pentahydrate); 80 mg 3b603 (Zinc oxide); 100 mg 3b502 (Manganese (II) oxide); 0.4 mg 3b801 (Sodium selenite); 1.6 mg 3b202 (Calcium iodate, anhydrous); 12,000 IU 3a672a (Vit. A); 40 mg 3a700 (Vit. E); 4.5 mg 3a711 (Vit. K_3_); 2.5 mg 3a821 (Vit. B_1_); 8 mg 3a825i (Vit. B_2_); 6 mg 3a831 (Vit. B_6_); 32 µg Vit. B_12_/Cyanocobalamin; 45 mg 3a315 (Niacinamid); 15 mg 3a841 (Calcium‐D‐pantothenate); 1.2 mg 3a316 (Folic acid); 50 µg 3a880 (Biotin); 550 mg 3a890 (Choline chloride).

^b^ Premix (for pullet diets) provides per kg diet: 0.91% crude ash; 0.20% Ca; 0.14% Na; 40 mg 3b103 (Iron (II) sulphate monohydrate); 15 mg 3b405 (Copper (II) sulphate pentahydrate); 80 mg 3b603 (Zinc oxide); 80 mg 3b502 (Manganese (II) oxide); 0.32 mg 3b801 (Sodium selenite); 1.6 mg 3b202 (Calcium iodate, anhydrous); 10,000 IU 3a672a (Vit. A); 25 mg 3a700 (Vit. E); 3 mg 3a711 (Vit. K_3_); 2.5 mg 3a821 (Vit. B_1_); 5 mg 3a825i (Vit. B_2_); 4 mg 3a831 (Vit. B_6_); 18.5 µg Vit. B_12_/Cyanocobalamin; 30 mg 3a315 (Niacinamid); 9 mg 3a841 (Calcium‐D‐pantothenate); 0.8 mg 3a316 (Folic acid); 300 mg 3a890 (Choline chloride).

During the trial, light was provided for 23 h for the first 2 days and was reduced to 16 h in the following weeks. Temperature program followed usual specifications for pullets. The animals were vaccinated against marek disease, salmonella, Newcastle disease and Infectious bronchitis.

The following evaluation of the laying hens in a subsequent laying trial is not part of the study presented here.

### 2.3 Measurements and Sample Collection

From hatching to Week 12, feed and residual feed were recorded weekly per pen to calculate the mean feed consumption.

The animals were weighed regularly using a common table scale (Sartorius, Sartorius AG, Göttingen, Germany, weighing accuracy ± 1 g). The results are presented analogous to the two feeding phases (Weeks 1–7 and Weeks 8–12).

After every 4 weeks, nine randomly selected animals per group (three females and six males, as the hens were scheduled for further observations during the laying phase) were sacrificed after individual body weight recordings. Blood samples were collected from three male animals for 25(OH)‐Vitamin D_3_ measurement.

At hatching, 24 chickens (6/genotype, male:female = 50:50) were slaughtered to obtain basal results for bone parameters. Afterwards, 72 animals (evenly distributed over all genotypes and diets) were slaughtered every 4 weeks to track bone development. For this, the tibiotarsi were preserved for further analyses (see Chapter 2.4). The livers were weighed individually.

### 2.4 Analyses

The relevant nutrient contents were analyzed in representative feed samples according to the methods of the VDLUFA (2017; see Table 3). The DM content of the feed samples was determined by weighing representative samples before and after drying at 103°C (VDLUFA number 3.1). Crude ash content was analyzed by weighing samples before and after combustion at 550°C in a muffle furnace (VDLUFA number 8.1). The total N content was measured by using the DUMAS method (VDLUFA number 4.1.2). The ether extract was measured using the soxhlet apparatus (VDLUFA number 5.1.1), and the crude fibre content was determined by sample treatment in diluted acid and alkaline solutions (VDLUFA number 6.1.1). Sugar was analyzed according to the Luff‐Schoorl method (VDLUFA number 7.1.2), while starch contents were determined by a polarimetrical method (VDLUFA number 7.2.1). After acid digestion, the optical emission spectrometry with inductively coupled plasma was used for mineral analysis (ICP‐OES Quantima; GBC Scientific Equipment Pty. Ltd., Melbourne, Vic, Australia; VDLUFA number 10.8.2).

**Table 3:** Analyzed nutrient contents in the experimental diets for chickens and pullets (g/kg DM).

Experimental diet	Chickens (Week of life 1–8)			Pullets (Week of life 9–12)		
Vit. D_3_ content (IU/kg)	300	1000	3000	300	1000	3000
DM (% as fed)	88.6	88.7	88.6	88.4	88.2	88.2
XA	70.6	71.7	72.3	66.1	66.0	64.7
XP	183.3	188.0	185.5	150.2	150.8	143.6
EE	40.6	42.2	38.8	33.9	34.5	33.2
XF	32.4	30.0	30.9	34.8	33.6	30.4
Sugar	36.5	39.1	39.2	38.2	43.1	39.9
Starch	514.2	512.5	514.3	564.2	567.8	566.7
ME (MJ/kg DM)	13.29	13.43	13.30	13.41	13.56	13.34
P	8.8	9.0	8.5	7.5	7.6	7.5
Ca	16.1	15.7	14.3	14.1	14.0	13.7
Mg	2.3	2.3	2.3	2.0	2.0	2.0
Na	3.6	3.8	3.6	4.1	4.2	4.3
Zn (mg/kg DM)	127.9	128.6	123.4	116.7	126.6	121.0

*Note:* ME (calculated, GfE Gesellschaft für Ernährungsphysiologie Ausschuss für Bedarfsnormen 1999: AME_N_ kJ/kg = 15.51 × XP, g/kg + 34.31 × EE, g/kg + 16.69 × Starch, g/kg + 13.01 × Sugar, g/kg).

Abbreviations: EE, ether extract; ME, metabolizable energy; XA, crude ash; XF, crude fibre; XP, crude protein.

After slaughter, various analyses were carried out with the tissue samples. Serum vitamin D concentrations, measured as 25(OH)‐Vitamin D_3_, were determined by high‐performance liquid chromatography with diode array detection (Sudwischer 2016; HPLC‐DAD; Shimadzu, Kyoto, Japan). First, a solid phase extraction (PhreeTM Phospholipid Removal 1 mL tube, Phenomenex) with acetonitrile/methanol was performed. The combined extracts were then evaporated at 40°C under a nitrogen flow. The dried residues were dissolved in methanol/distilled water (40/60) and filtered (syringe filters, 13 mm, 0.45 µm, PVDF, amchro GmbH) before HPLC injection. The HPLC parameters were as follows: oven temperature: 40°C, autosampler temperature: 4°C, column: Synergi 4 µ Hydro‐RP 80 A; 4 µm; 250 × 3.0 mm, gradient elution with acetonitrile (mobile phase A) and methanol/distilled water (40/60) (mobile phase B), DAD at 265 nm. Quantification limit was 15.1 µg/L, and detection limit was 6.4 µg/L.

**Figure 2:**
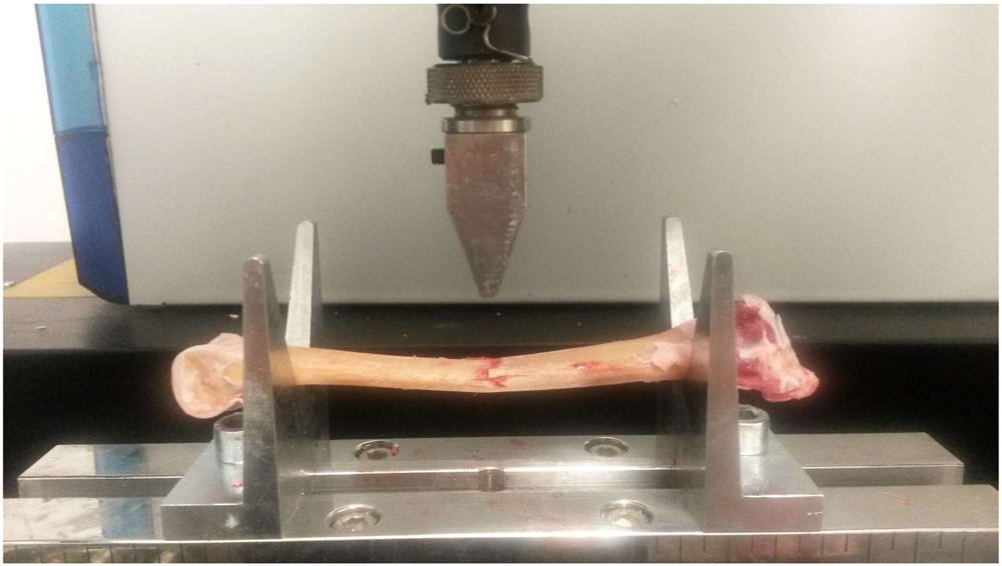
Measuring of bone breaking strength. [Color figure can be viewed at wileyonlinelibrary.com]

The right tibiotarsus was weighed, and the volume was determined by water displacement of the bone in a beaker glass. Calcium and phosphorus contents were then determined in freeze‐dried samples by optical emission spectrometry (ICP‐OES Quantima; GBC Scientific Equipment Pty. Ltd., Melbourne, Vic, Australia; VDLUFA number 10.8.2). The left tibiotarsus was used to determine both length (between joint surfaces) and diameter (centre of tibiotarsus) using a caliper. Bone breaking strength (Model 4301, Instron, High Wycombe, United Kingdom, cf. Habig and Distl 2013) was measured afterwards. For this purpose, the bones were placed on two support surfaces, and weight was applied vertically to the centre of the bone until fracture (Figure 2).

### 2.5 Statistical Analyses

The software SAS (9.4, SAS Institute Inc., Cary, NC, USA) was used for statistical analyses. The Kruskal–Wallis test was used to statistically evaluate vitamin D concentrations in the serum of day‐old chickens. Statistical analyses concerning feed consumption, body weight development, liver weights, serum 25(OH)‐Vitamin D_3_ concentrations as well as bone parameters during the trial were performed using the PROC MIXED procedure of SAS (version 9.4; SAS Institute Inc., Cary, NC) with a restricted maximum likelihood model. The covariance structure was chosen according to the corrected Akaike's information criterion, where appropriate, with covariables as means of the values obtained on the first day of life. An adjusted Tukey–Kramer test was applied as a post hoc procedure. *p*‐values < 0.05 were considered significant.

## 3 Results

### 3.1 Feed Consumption

**Figure 3:**
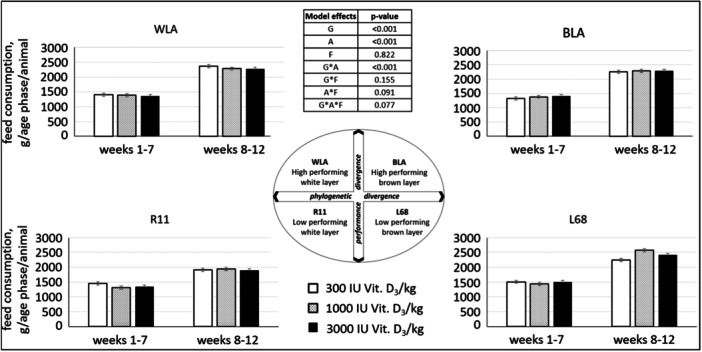
Feed consumption per animal (g/animal), depending on genotype, age and experimental diet (LSMeans ± SE, *N* = 60; G: genotype, A: age, F: feed).

Feed consumption per animal, distinguished into chicken (Weeks 1–7) or pullet (Weeks 8–12) phases, differed between genotypes and was influenced by the age of the animals (Figure 3). The interaction between genotype and age was significant: In all genotypes, animals consumed more feed in the second age phase (Weeks 8–12) than in Weeks 1–7 (*p* < 0.001). Moreover, feed intake was higher in Weeks 8–12 for L68 compared to R11 and WLA, as well as for BLA compared to R11 (*p* < 0.001).

### 3.2 Body Weight Development

**Figure 4:**
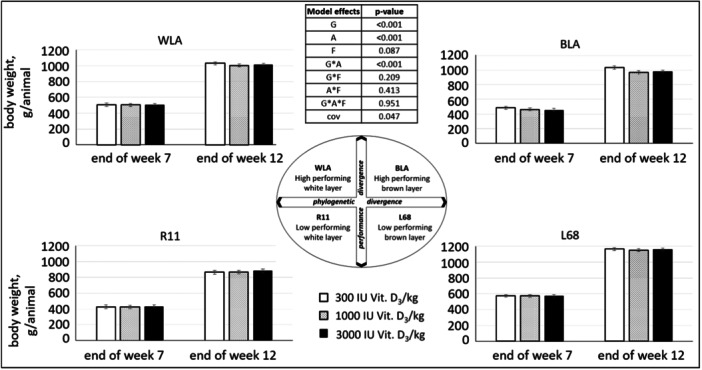
Body weight development (g) depending on age, genotype and dietary vitamin D_3_ content in male and female chickens (LSMeans ± SE, *N* = 60; G: genotype, A: age, F: feed, cov: covariable/body weight at 1st day of life).

The average body weight of the animals at the end of the above‐mentioned feeding periods, based on group weighing and divided by the number of animals in the group, is presented in Figure 4.

Over the course of the trial, body weight was influenced by genotype and age. At hatching, the weight of the chickens did not differ regarding genotype (*p* > 0.9; covariable for further calculations: means of WLA/BLA/R11/L68 = 38.4/39.6/31.4/33.6 g), but later on L68 showed the highest body weights, followed by WLA, BLA and R11. Body weight increased significantly between feeding phases in every genotype.

### 3.3 Blood Parameters

**Figure 5:**
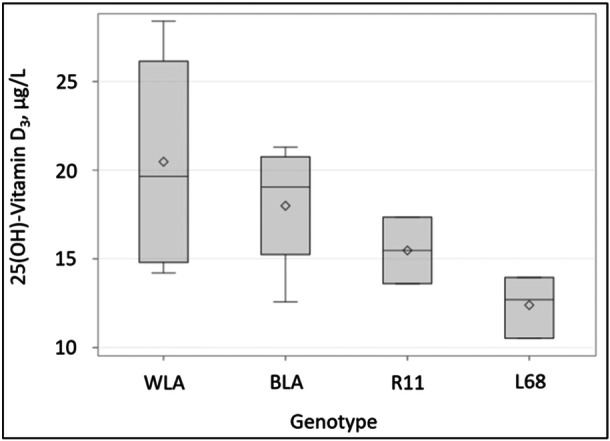
Serum vitamin D status at hatch of chicken, depending on genotype (*N* = 13; means ± SD, rhombus = mean value; line = median. The vertical boxes show the range between the 25th and 75th percentiles, with whiskers extending to 2.5 of the interquartile range).

Serum vitamin D status at hatch (Figure 5) differed numerically but not significantly (*p* = 0.157).

**Figure 6:**
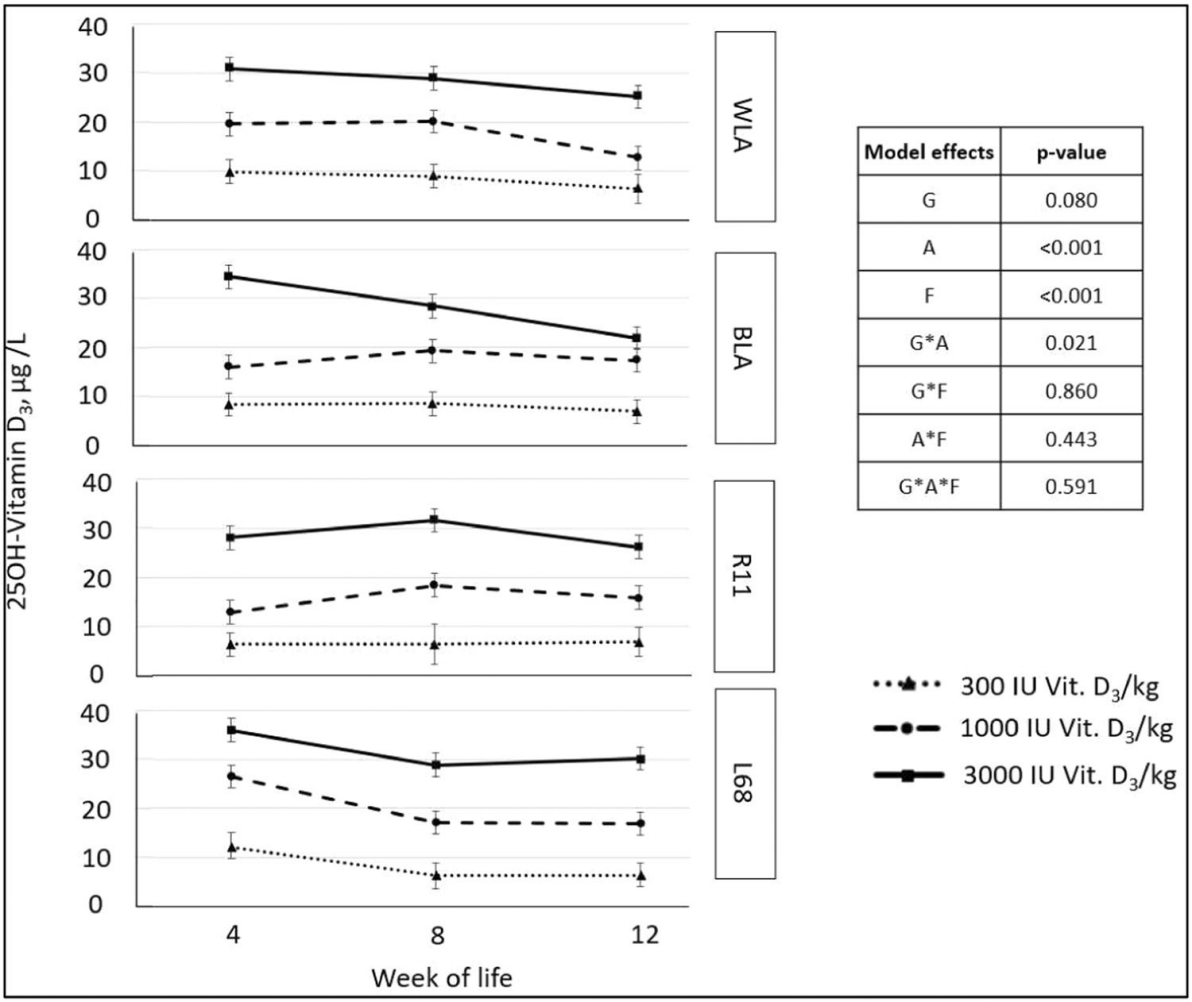
Serum vitamin D status of male chickens (*n* = 3 per group and measurement, with seven non‐evaluable samples) depending on genotype, age and experimental diet (LSMeans ± SE; G: genotype, A: age, F: feed).

Figure 6 presents the results of blood samples collected from 3 males per group after 4, 8 and 12 weeks of rearing. The targeted differences regarding vitamin D_3_ supply are clearly reflected in the serum. Significant differences could be observed with respect to experimental diet (values of each diet significantly different from all other diets, *p* < 0.001) and age of the animals (higher values in Week 4 than in Week 12, *p* < 0.001 and higher values in Week 8 than in Week 12, *p* = 0.05), as well as the interaction of genotype x age (L68, Week 4, had higher serum 25(OH)‐vitamin D_3_ concentrations than R11 in Week 4, with LSMeans of 24.8 and 15.8 µg/L, *p* < 0.001).

### 3.4 Bone Characteristics and Liver Weights

The bone parameters (tibiotarsus) of the day‐old chickens are summarized in Table 4. At hatching, relative tibiotarsal weight was higher in L68 (LSMean = 0.131 g/100 g body weight) and R11 (LSMean = 0.136 g/100 g body weight) than in WLA (LSMean = 0.105 g/100 g body weight) and BLA (LSMean = 0.103 g/100 g body weight), with *p* ≤ 0.003. Diameter, length and volume as well as bone‐breaking strength did not differ significantly at hatching.

**Table 4:** Relative tibiotarsal weight, diameter, length and volume of the tibiotarsus, bone‐breaking strength as well as calcium and phosphorus content of the tibiotarsus in day‐old chickens of different genotypes (LSMeans ± SE, *n* = 3 per genotype and sex^a^).

Genotype	Sex	Relative tibiotarsal weight (g/100 g body weight)	Diameter (cm)	Length (cm)	Volume (cm³)	Bone breaking strength (*N*)	Ca content (g/kg)	P content (g/kg)
WLA	f	0.101 ± 0.01	0.1 ± 0.02	2.6 ± 0.12	0.2 ± 0.02	4.7 ± 0.6	164.0 ± 4.0	69.9 ± 2.2
WLA	m	0.109 ± 0.01	0.1 ± 0.02	2.8 ± 0.12	0.2 ± 0.02	5.0 ± 0.6	157.7 ± 4.0	65.9 ± 2.2
BLA	f	0.107 ± 0.01	0.2 ± 0.02	2.6 ± 0.15	0.2 ± 0.02	4.5 ± 0.7	157.3 ± 4.0	68.7 ± 2.2
BLA	m	0.099 ± 0.01	0.1 ± 0.02	2.7 ± 0.12	0.2 ± 0.02	4.3 ± 0.6	153.9 ± 4.0	66.2 ± 2.2
R11	f	0.136 ± 0.01	0.2 ± 0.02	2.5 ± 0.12	0.2 ± 0.02	5.0 ± 0.7	152.6 ± 4.0	66.6 ± 2.2
R11	m	0.135 ± 0.01	0.1 ± 0.02	2.7 ± 0.12	0.2 ± 0.02	6.0 ± 0.7	155.1 ± 4.0	65.2 ± 2.2
L68	f	0.123 ± 0.01	0.1 ± 0.02	2.7 ± 0.12	0.2 ± 0.02	4.7 ± 0.6	161.7 ± 4.0	69.1 ± 2.2
L68	m	0.139 ± 0.01	0.2 ± 0.02	2.7 ± 0.12	0.2 ± 0.02	5.0 ± 0.6	148.3 ± 4.0	66.0 ± 2.2

Model effects	*p*‐values						
G	< 0.001	0.828	0.802	0.266	0.469	0.346	0.797
S	0.455	1.000	0.431	0.350	0.406	0.090	0.090
G × S	0.245	0.468	0.898	0.665	0.854	0.297	0.945

Abbreviations: f, female; G, genotype; m, male; S, sex.

^a^ *n* = 3 except bone breaking strength (*n* = 2 for BLA female, R11 female and R11 male) and except length (*n* = 2 for BLA female).

Calcium and phosphorus contents in the tibiotarsus were also not significantly different between sexes or genotypes at hatching.

**Figure 7:**
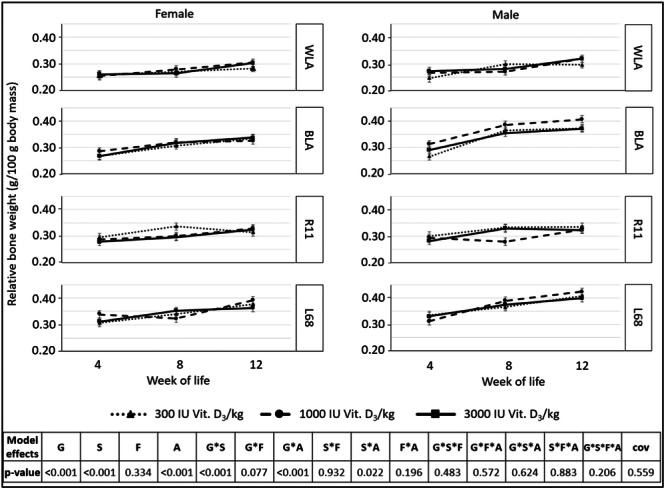
Relative tibiotarsal weight (g tibiotarsus/100 g body weight) of chickens, depending on genotype, age, sex and dietary vitamin D_3_ content (LSMeans ± SE; *n* = 3 per genotype and sex; G: genotype, S: sex, A: age, F: feed, cov: covariable/relative tibiotarsal weight at 1^st^ day of life).

Relative tibiotarsal weight changed during rearing up to 12 weeks (Figure 7). Genotype, sex and age had an impact on relative tibiotarsal weight (interactions G × A and S × A): Older chickens had higher relative tibiotarsal weights (*p* < 0.001), as did male animals in comparison to female ones (LSMeans for male vs. female: 0.328 vs. 0.309 g/100 g body weight, *p* < 0.001). The descending order of genotypes was L68 > BLA > R11 > WLA (LSMeans: 0.357/0.329/0.306/0.281 g/100 g body weight, *p* ≤ 0.023).

**Figure 8:**
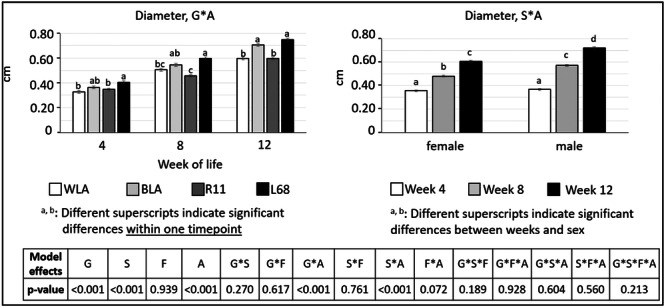
Diameter of tibiotarsi in chickens depending on genotype, age, sex and diet (LSMeans ± SE; *n* = 3 per genotype and sex; G: genotype, S: sex, A: age, F: feed).

**Figure 9:**
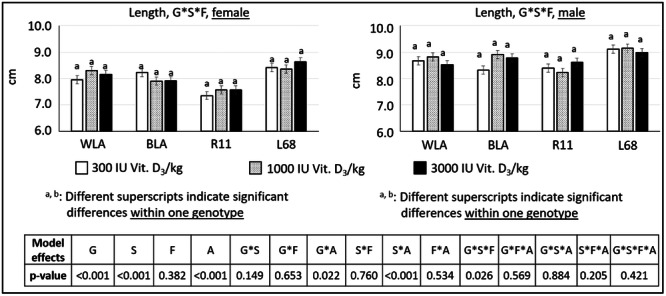
Length of tibiotarsi in chickens depending on genotype, age, sex and diet (LSMeans ± SE; *n* = 3 per genotype and sex; G: genotype, S: sex, A: age, F: feed).

**Figure 10:**
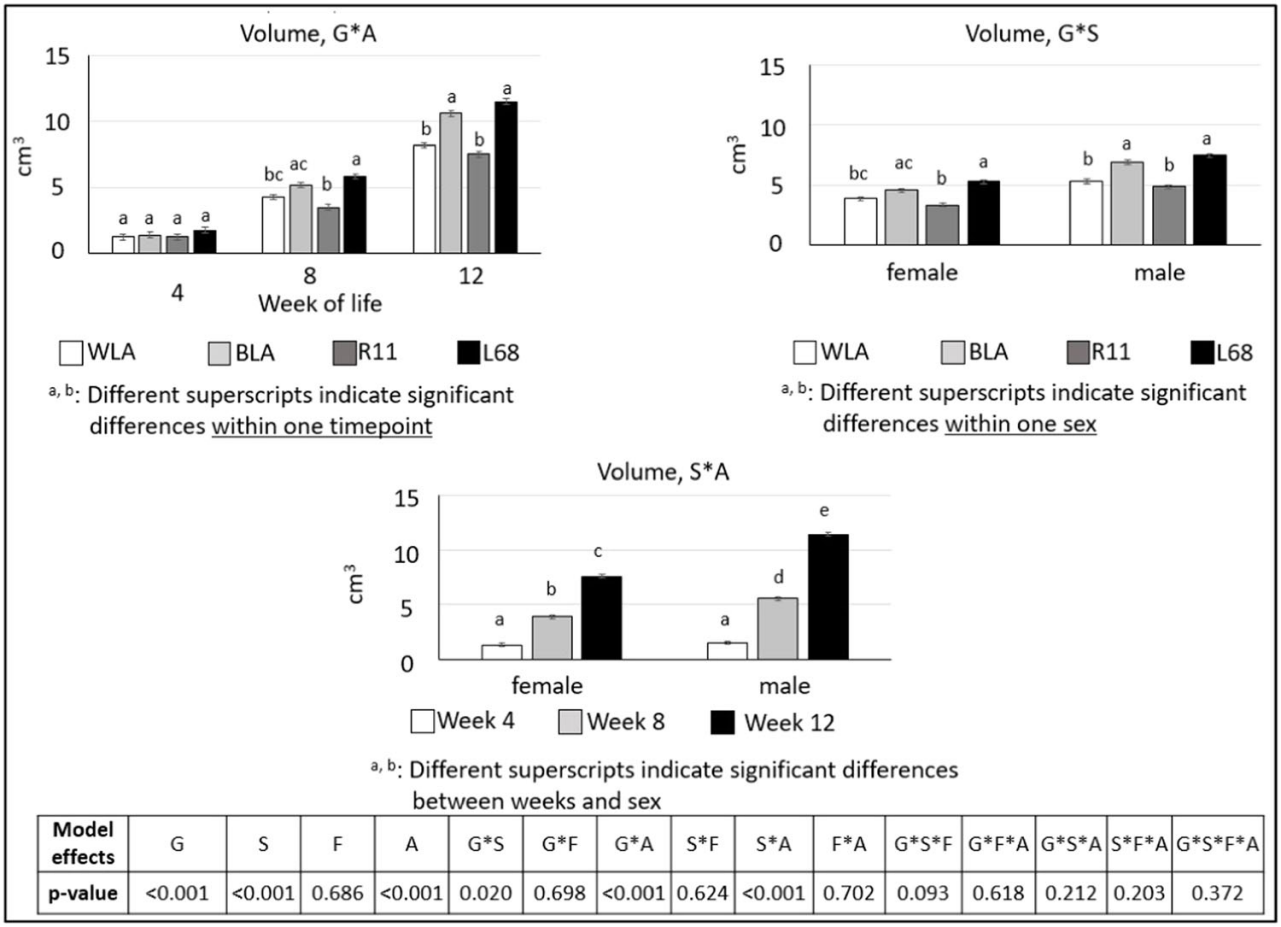
Volume of tibiotarsi in chickens depending on genotype, age, sex and diet (LSMeans ± SE; *n* = 3 per genotype and sex; G: genotype, S: sex, A: age, F: feed).

There were also some significant differences regarding diameter, length and volume of the tibiotarsus. All values are shown in tabular form (Table 5), but significant interactions are presented in Figures 8, 9, 10.

**Table 5:** Thickness, length and volume of the tibiotarsus in chickens of different genotypes depending on dietary vitamin D3 content and age (LSMeans; *N* = 216).

		Week of life 4			Week of life 8			Week of life 12		
		300 IU	1000 IU	3000 IU	300 IU	1000 IU	3000 IU	300 IU	1000 IU	3000 IU
Diameter (cm)										
WLA	m	0.4	0.3	0.4	0.5	0.5	0.6	0.7	0.6	0.7
	f	0.3	0.3	0.3	0.4	0.5	0.5	0.6	0.5	0.5
BLA	m	0.3	0.4	0.4	0.6	0.6	0.6	0.8	0.8	0.8
	f	0.4	0.4	0.3	0.5	0.5	0.5	0.7	0.6	0.6
R11	m	0.4	0.4	0.3	0.5	0.5	0.5	0.7	0.6	0.6
	f	0.4	0.4	0.3	0.4	0.4	0.5	0.6	0.6	0.5
L68	m	0.4	0.4	0.4	0.6	0.7	0.7	0.8	0.8	0.8
	f	0.4	0.4	0.4	0.6	0.5	0.5	0.7	0.7	0.8
Length (cm)										
WLA	m	5.5	5.9	5.6	9.0	8.9	8.6	11.5	11.6	11.4
	f	5.6	5.3	5.8	8.1	8.5	8.1	10.1	11.1	10.6
BLA	m	5.0	5.8	5.6	8.4	9.2	9.0	11.6	11.8	11.8
	f	5.4	5.3	5.3	8.3	7.8	8.1	11.0	10.6	10.4
R11	m	5.5	5.7	5.7	8.1	8.5	8.7	11.6	10.5	11.5
	f	5.1	5.3	5.4	7.4	7.3	7.3	9.6	10.1	10.0
L68	m	6.2	5.8	5.8	8.7	9.5	9.2	12.5	12.1	12.0
	f	5.6	6.0	6.2	8.5	8.5	8.7	11.1	10.5	11.1
Volume (cm³)										
WLA	m	1.4	1.5	1.3	5.1	4.8	5.0	9.9	10.1	8.9
	f	1.3	1.0	1.3	3.7	4.0	3.3	5.7	7.0	7.8
BLA	m	1.1	1.9	1.7	5.4	6.7	6.7	12.7	13.4	12.8
	f	1.3	1.4	1.3	4.3	3.7	4.4	8.9	8.1	7.9
R11	m	1.3	1.4	1.5	3.8	4.0	4.6	10.2	7.9	9.2
	f	1.1	1.2	1.2	3.3	2.8	2.7	5.8	6.2	6.0
L68	m	2.1	1.7	1.8	6.2	7.1	7.0	14.0	13.7	13.9
	f	1.6	1.7	1.9	5.0	4.9	5.0	9.9	7.8	10.0
*p*‐value										
		G	S	F	A	G × S	G × F	G × A	S × F	S × A
D		< 0.001	< 0.001	0.939	< 0.001	0.270	0.617	< 0.001	0.761	< 0.001
L		< 0.001	< 0.001	0.382	< 0.001	0.149	0.653	0.022	0.760	< 0.001
V		< 0.001	< 0.001	0.686	< 0.001	0.020	0.698	< 0.001	0.624	< 0.001
		F × A	G × S × F	G × F × A	G × S × A	S × F × A	G × S × F × A			
D		0.072	0.189	0.928	0.604	0.560	0.213			
L		0.534	0.026	0.569	0.844	0.205	0.421			
V		0.702	0.093	0.618	0.212	0.203	0.372			

Abbreviations: A, age; D, Diameter; F, feed; G, genotype; L, Length; S, sex; V, Volume.

Significant differences concerning *diameter* of tibiotarsi in genotype × age were contributed to increasing values within one genotype from one sample collection to the next, as well as to some differences between genotypes: Bone diameter was higher in BLA than in R11 in Weeks 8 and 12 (*p* < 0.001). Bone diameter was also higher in BLA than in WLA in Week 12 (*p* < 0.001). Bone diameter in L68 was higher than in R11 and in WLA in all measurements (*p* ≤ 0.024 for R11 and *p* < 0.001 for WLA, respectively).

Sex × age differences in bone diameter were evident in all comparisons (*p* < 0.001), except between males and females in Week 4 (*p* = 0.929) and between males in Week 8 and females in Week 12 (*p* = 0.051).

*Length* was influenced by genotype, sex and age. Effects due to different diets in the same genotype and sex within one timepoint could not be observed.

Significant interactions regarding *volume* of tibiotarsi are displayed in Figure 10. Bone volume was influenced by age and genotype as well as by sex and age in an interactive manner. While all genotypes demonstrated similar bone volumes at 4 weeks of age, a more pronounced increase was observed in BLA and L68 in Weeks 8 and 12 compared to WLA and R11.

The volumes of females and males did not differ after 4 weeks of life, but in Weeks 8 and 12.

**Figure 11:**
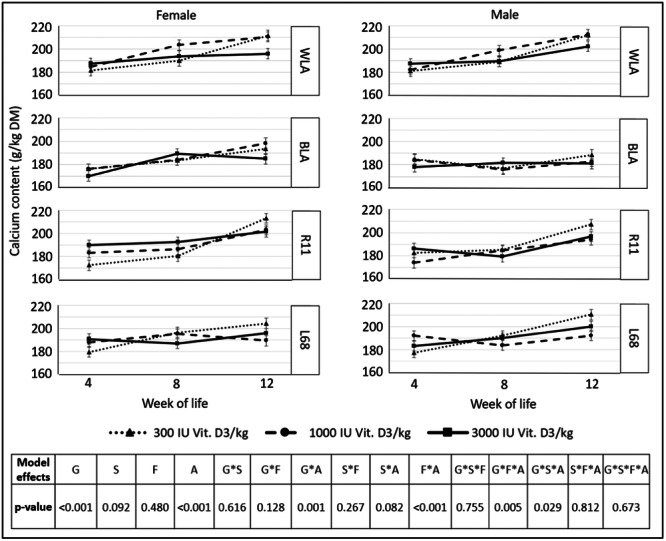
Ca content (g/kg DM) of tibiotarsus, depending on genotype, age, sex and dietary vitamin D_3_ content (LSMeans ± SE; *N* = 216; G: genotype, S: sex, A: age, F: feed).

**Figure 12:**
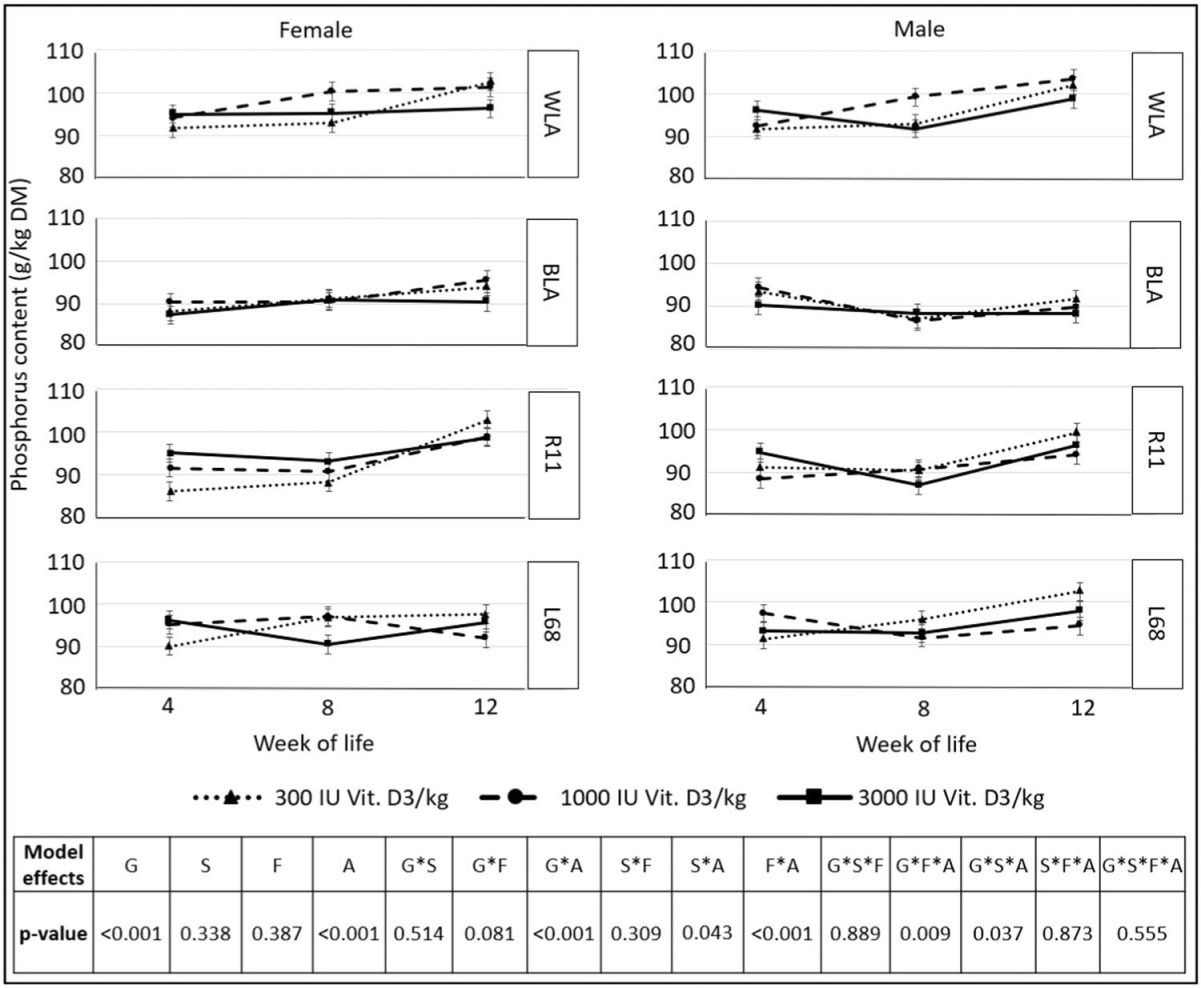
P content (g/kg DM) of tibiotarsus, depending on genotype, age, sex and dietary vitamin D_3_ content (LSMeans ± SE; *N* = 216; G: genotype, S: sex, A: age, F: feed).

Mineralization of the tibiotarsus, in terms of *calcium and phosphorus content*, is shown in Figures 11 and 12.

Significant interactions for the Ca content regarding genotype × feed × age were related to different measurement times (higher Ca contents in older animals, *p* < 0.001), or genotypes (differences between all genotypes (*p* ≤ 0.001), except L68 versus R11 (*p* = 0.577) and L68 versus WLA (*p* = 0.062)).

Ca content in the tibiotarsus also showed significant interactions regarding genotype × sex × age, but without significant differences between male and female animals of the same genotype at any timepoint.

Regarding the P content of the tibiotarsus, it was analyzed that genotype × feed × age also showed a significant interaction, but there were no differences within 1 week regarding different experimental diets in any genotype.

**Figure 13:**
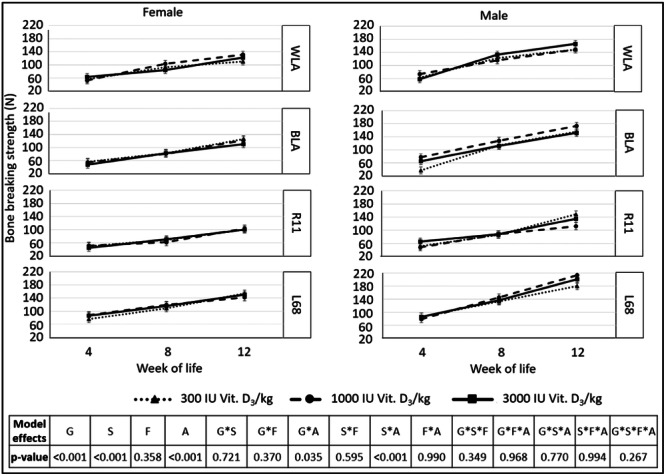
Bone breaking strength of tibiotarsus (N) of male and female chickens of different genotypes, ages and fed different dietary contents of vitamin D_3_, *n* = 3 chickens per genotype, sex, diet and week of life (LSMeans ± SE; *N* = 216; G: genotype, S: sex, A: age, F: feed).

No significant effect of dietary vitamin D_3_ content on bone‐breaking strength of tibiotarsus could be measured, but age × sex as well as genotype × age did have an impact (Figure 13).

Bone breaking strength increased between sampling times in each genotype (*p* ≤ 0.004).

With respect to sex × age, bone‐breaking strength of the tibiotarsus did not differ between male and female animals in Week 4 (*p* = 0.846), but in Weeks 8 and 12, where breaking strength for tibiotarsi was higher in male animals (*p* < 0.001). In addition, breaking strength increased significantly from Week 4 to Week 8 to Week 12 in both male and female animals (*p* < 0.001).

**Figure 14:**
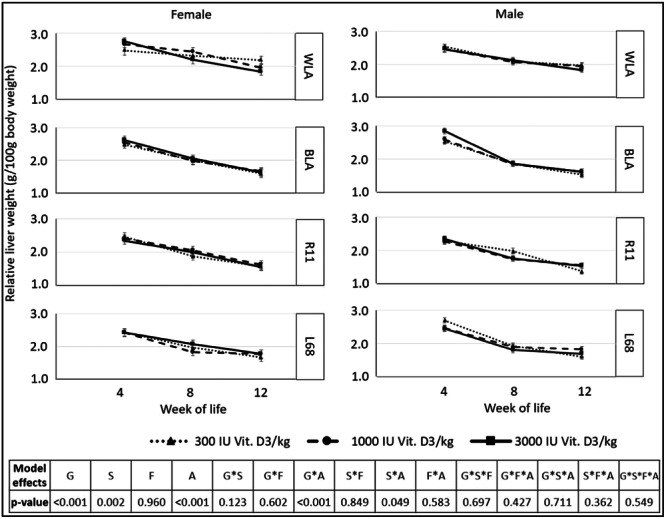
Relative liver weight of male and female chickens of different genotypes, ages and fed different dietary contents of vitamin D_3_, *n* = 3 chickens per genotype, sex, diet and week of life (LSMeans ± SE; *N* = 324; G: genotype, S: sex, A: age, F: feed).

The liver weights of slaughtered chickens were evaluated by calculating the relative organ weight, by relating the organ weight to the body mass (Figure 14).

Genotype × age interactions were significant: Within a genotype, relative liver weight decreased from each timepoint to the next (*p* ≤ 0.05). Sex and age also showed significant differences: Relative liver weights decreased from one timepoint to the next in females (LSMeans: 2.5/2.06/1.7 g/100 g body weight, *p* < 0.001), as well as in males (LSMeans: 2.5/1.9/1.7 g/100 g body weight, *p* < 0.001). In Week 8, relative liver weights also differed between females (with higher relative liver weights) and males (*p* = 0.004).

## 4 Discussion

A rearing trial was conducted with chickens of different genotypes at three different vitamin D_3_ contents in the complete diet within the EU legislation to assess whether a different genotype or a different performance should be considered regarding the dietary vitamin D_3_ supply during the rearing of laying hens.

*Feed consumption* showed some significant differences between the genotypes, which could be due to the different body weight development and, therefore, different capacity for DM intake (Cheeke and Dierenfeld 2010; McDonald et al. 2011). Lieboldt et al. (2015) observed in the same genotypes that R11 had the lowest feed intake in Weeks 1–12, followed by WLA and BLA/L68 in Weeks 9–12. In this study, R11/BLA/WLA/L68 consumed 273.8/303.3/307.0/324.7 g feed/animal/week (LSMeans, *p* ≤ 0.010 for each comparison).

Adhikari et al. (2020) observed an impact of different dietary vitamin D contents and vitamin D isoforms (vitamin D_2_, vitamin D_3_ or 25‐OHD) on feed intake in a 6‐week lasting trial with laying hens (57 weeks old at the beginning of the trial), whereby a diet with 3000 IU Vit. D_3_ served as control. For example, hens fed a diet with an additional 9000 IU Vit. D_2_/kg (additional to the control of 3000 IU Vit. D_3_/kg) showed a lower feed intake than hens fed a diet with an additional 3000 IU Vit. D_2_/kg in Weeks 1, 2, 3, 5 and 6. In Week 6, there was also a significant lower feed intake for the group fed an additional 9000 IU Vit. D_2_/kg in comparison to the control group. Adhikari et al. were unable to provide an explanation for these and further findings regarding feed intake. In the present study, comparably high dietary vitamin D contents or other isoforms of vitamin D were not part of the experiment, so no negative effect of dietary vitamin D_3_ supply on feed intake could be observed. Geng et al. (2018) used three different diets for laying hens (Jinghong laying hens, 32 weeks old, 10 weeks feeding trial) containing 0, 500, 1500 or 3000 IU Vit. D_3_/kg. No differences in feed intake were seen for diets with 500, 1500 and 3000 IU Vit. D_3_/kg, but a higher feed intake for the diet without vitamin D_3_ supplementation. The results are also in good agreement with the findings in the presented study.

*Vitamin D status* in blood samples differed at hatch numerically, but not significantly. Vitamin D supply of laying hens influences the vitamin content of egg yolk and the hatched chicken (Bethke et al. 1936; Stevens and Blair 1985; NRC 1994). In this study, all parent animals received the same diet, so the numeric variation in the analyzed vitamin D concentrations in the serum of the hatched chickens in this study is actually insignificant for further evaluation.

During the rearing period, the 25(OH)‐vitamin D_3_ contents in serum followed the different dietary vitamin D_3_ contents (Figure 6). After absorption, vitamin D enters the bloodstream and is distributed throughout the body (McDonald et al. 2011), with serum being one of the tissues with the highest concentrations (Leeson and Summers 2001). The genotype‐dependent differences in feed intake reported above were not related to the 25(OH)‐vitamin D_3_ contents in serum so groups and genotypes within dietary treatments are comparable.

Geng et al. (2018) fed 32‐week‐old laying hens with a diet containing 3000 IU Vit. D_3_/kg and reported a mean serum concentration of 27.39 25(OH)‐Vitamin D_3_ ± 2.97 µg/L, which is in good agreement with the observed results of the present study (LSMeans ± SE: 29.2 ± 0.715 µg/L for the diet containing 3000 IU Vit. D_3_/kg). Interestingly, hens fed a vitamin D‐deficient diet (0 IU Vit. D_3_/kg) in the study by Geng et al. (2018) showed serum concentrations of 7.60 25(OH)‐Vitamin D_3_ ± 2.85 µg/L, which is close to the 300 IU Vit. D_3_‐group in the present study (LSMeans ± SE: 7.8 ± 0.812 µg/L for the diet with 300 IU Vit. D_3_/kg). An undersupply of pullets in the present study is unlikely, as hens from the same study, reported by Jansen et al. (2020), showed no significant differences in bone mineral density or bone‐breaking strength between groups fed with 300 or 3000 IU Vit. D_3_/kg after 68 weeks of life.

*Body weight development* differed between genotypes, which is in line with the results of other studies (Jansen et al. 2020; Lieboldt et al. 2015).

The dietary treatment had a significant effect on body weight only after the 12th week of life, with heavier chickens fed the diet with 300 IU Vit. D_3_/kg than those fed the diet with 1000 IU Vit. D_3_/kg. However, as animals on the diet with 3000 IU Vit. D_3_/kg showed intermediate body weights, this is likely to be an effect other than the dietary vitamin D_3_ content. Wen, Livingston, and Persia (2019) used diets with different vitamin D_3_ contents for rearing pullets and did not observe any differences in body weight in Week 17 up to 35,014 IU Vit. D_3_/kg diet.

*Tibiotarsus characteristics* differed among groups: Even on the first day of life, relative bone weight was higher in L68 and R11 than in WLA and BLA, although thickness, length and volume did not differ.

Statistical evaluation of the *diameter* revealed differences, especially regarding genotype (L68 > BLA > WLA and R11). This is in agreement with the findings of Jansen et al. (2020), who found the same order in laying hens of the same genotypes, also without significant differences between WLA and R11. Phenotypic differences, especially in brown and white layer lines, seem to be responsible for these findings, as suggested by Jansen et al. (2020).

Bone *lengths* were examined for differences that could be due to dietary vitamin D content, as an impact of genotype and sex seems to be evident (compare Jansen et al. 2020). Significant three‐way interactions of genotype × sex × feed did not involve the same genotype and sex so an impact of dietary vitamin D_3_ content could not be validated in this study.

Tibiotarsus *volume* was also not influenced by dietary vitamin D content in this study.

The study by Habig et al. (2017), in which the same genotypes were reared in different housing systems, confirms that the dimensions of tibiotarsus are mainly influenced by genotype.

*Calcium and phosphorus contents* of the tibiotarsus were not affected by dietary vitamin D_3_ contents, but interactions with genotype, sex and age were partially significant. Jansen et al. (2020) also reported different bone mineral densities associated with different genotypes in laying hens. Wen, Livingston, and Persia (2019) reported an increase in bone mineral content between 17‐week‐old pullets fed 1681 versus 8348 IU Vit. D_3_/kg diet. The range of recommended and permitted dietary vitamin D_3_ contents in the EU does not appear to be variable enough to detect possible effects of vitamin D_3_ on measured bone parameters in these genotypes. In contrast to this, Liermann et al. (2023) reported beneficial effects on calcium and phosphorus homoeostasis in high‐performing WLA and BLA during the early laying period when they were fed 3000 instead of 300 IU Vit. D_3_/kg diet.

*Breaking strength* of the tibiotarsus was influenced by genotype and sex, but not by dietary vitamin D_3_ content. Habig et al. (2017) reported higher tibial bone‐breaking strength in L68 compared to BLA in the 74th week of life. The authors assumed that lower‐performing genotypes may have a higher bone‐breaking strength due to changes in the histological structure of the bone. As the animals in the present study did not reach sexual maturity, this hypothesis can neither be accepted nor rejected, but similar results were observed in the present study regarding the low‐performing L68 and R11 compared to their high‐performing counterparts BLA and WLA.

Jansen et al. (2020), who also found differences between the genotypes WLA, R11, BLA and L68 in laying hens, reported that breaking strength of bones was mainly influenced by bone mineralization. In this study, the Ca content in L68 was higher than in BLA (LSMeans: 193.8 vs. 184.1 g/kg DM, *p* = 0.004), so that this theory still deserves consideration, even if the influence of the dietary vitamin content remains questionable.

In this study, only legally permitted vitamin D contents were tested. As no clinical health problems were observed in this study, there does not seem to be a need to adjust current nutrient recommendations. Moderate oversupply of vitamin D (below toxic levels) may be associated with beneficial effects on bone metabolism, as Whitehead et al. (2004) could prove for young broilers. Safe upper limits for vitamin D_3_ have been established because vitamin D may have toxic effects in case of oversupply (EFSA Panel on Additives Products or Substances used in Animal Feed 2014). However, an imminent increase in the legal limit at the EU level is not likely.

## 5 Conclusions

The observed high and moderate‐performing layer lines differed in some basic production parameters (feed intake, body weight development, bone morphology) already in the first weeks of rearing. The legally permitted (EU legislation) dietary vitamin D_3_ contents in the range of 300–3000 IU Vit. D_3_/kg diet did not result in any advantage or disadvantage for the animals in terms of performance or bone health under the rearing conditions described. A specific dietary recommendation for vitamin D_3_ supply during the rearing of low or moderate‐performing layers does not appear to be necessary on the basis of the present results. For sustainability reasons, it may be prudent not to insist on achieving the maximum permitted dietary vitamin D contents, although safety margins in the complete feed are always advisable. Genotype‐related differences in bone characteristics are evident and should be considered, especially in the context of the politically demanded transformation of agriculture.

## Ethics Statement

The authors confirm that the ethical policies of the journal, have been adhered to and the appropriate ethical review committee approval has been received (Lower Saxony State Office for Consumer Protection and Food Safety, LAVES, Germany; file number 33.92 42502‐04‐13/1186 and 33.19 42502‐04‐15/1816). The authors confirm that they have followed EU standards for the protection of animals.

## Conflicts of Interest

The authors declare no conflicts of interest.

## Data Availability

The data that support the findings of this study will be openly available in zenodo at https://zenodo.org/ after publication of the paper.
